# Determinants of child stunting in the dryland area of East Nusa Tenggara Province, Indonesia: insights from a national-level survey

**DOI:** 10.25122/jml-2023-0313

**Published:** 2024-02

**Authors:** Intje Picauly, Anak Agung Ayu Mirah Adi, Eflita Meiyetriani, Majematang Mading, Pius Weraman, Siti Fadhilatun Nashriyah, Daniela Leonor Adeline Boeky, Varry Lobo, Asmulyati Saleh, Jane Austen Peni, Ahmad Thohir Hidayat, Marni Marni

**Affiliations:** 1 Department of Public Health, Nusa Cendana University, Kupang, Indonesia; 2 Department of Nutrition, Kupang Ministry of Health Health Polytechnic, Kupang, Indonesia; 3 SEAMEO RECFON, Center for Regional Nutrition Studies, Jakarta, Indonesia; 4 Institute of Research and Development Waikabubak, Sumba Barat, Indonesia

**Keywords:** stunting, household, dry land area, low birth weight

## Abstract

Stunting remains a critical public health issue in Indonesia, particularly in the province of East Nusa Tenggara. This region, characterized by its archipelagic dryland geography, has reported the highest prevalence of stunting among children under five from 2007 to 2021. The study aimed to examine the relationship between various characteristics of children under five and household factors with the occurrence of stunting. This observational study, with a cross-sectional design, used secondary data from the 2021 Indonesian Nutrition Status Survey, covering 7,835 children under five. We analyzed the data to identify patterns and relationships, using univariate analysis to display percentage distributions and bivariate analysis through multiple binary logistic regression tests. The results of the multiple logistic regression test showed that indicators of family characteristics such as age, gender, low birth weight, body length, possession of birth certificates, and receiving complementary feeding were all related to stunting. Additionally, household factors such as toilet type, National Health Insurance coverage, ownership of a Prosperous Family Card, and residential area were significant determinants. Factors contributing to stunting in dryland areas include a range of elements from both family characteristics—such as age, gender, birth certification, low birth weight, and initial body length, to the introduction of supplementary feeding—and household indicators, including the use of specific types of latrines (*Plengsengan* and *Cemplung* types without covers), health insurance coverage, possession of Prosperous Family Cards, and the family's residential area.

## INTRODUCTION

Stunting remains a significant challenge in global health, characterized by chronic malnutrition resulting from long-term inadequate nutritional intake, causing children to be significantly shorter compared to peers of the same age. Nusa Tenggara Timur (NTT) has the highest stunting prevalence rate among the 34 provinces in Indonesia, with a rate of 20.9% as of 2021 [1]. In addition, the prevalence rate is still above the national and the World Health Organization (WHO) threshold, which is 20%.

The development and growth of children experiencing stunting are significantly impacted by various factors within their family and household environment, such as inadequate access to nutritious meals, not receiving exclusive breastfeeding, and frequent exposure to infectious diseases [2,3]. These elements are embedded within the broader social context, including political and economic influences, healthcare provision, educational opportunities, socio-cultural norms, agricultural practices, food availability, and access to clean water, sanitation facilities, and a hygienic living environment.

This research aimed to investigate the relationship between various characteristics of children under five and household factors with the incidence of stunting. Furthermore, we aimed to inform the development of strategic policies and effective intervention measures to reduce the prevalence of stunting in the NTT province, addressing a key issue in the public health domain and contributing to the broader efforts to improve child health and development.

## MATERIAL AND METHODS

### Study design and participants

This study utilized secondary district-representative data from the Indonesian Nutritional Status Survey (INSS) 2021, focusing on children under five. This study was part of a larger study titled 'Nutritional Status of Children Age 0-59 Months in Indonesia' conducted by the Indonesian Ministry of Health. The INSS, a national-scale survey, aims to determine developments in the nutritional status of children under five (stunting, wasting, and underweight) at the national, provincial, and district/city levels. The study, conducted in 2019, was carried out by the Health Research and Development Agency of the Ministry of Health in collaboration with the Central Bureau of Statistics and supported by the Secretariat of the Vice President of the Republic of Indonesia. Currently, the implementation of this survey is mandated by the Presidential Decree No. 72 of 2021 where the Ministry of Health is responsible for publishing district/city stunting prevalence data annually. The survey results also form the basis for the Ministry of Finance to determine district/city Regional Incentive Funds as well as material for evaluating the implementation of nutrition interventions, both specific and sensitive, carried out by the government at the central and regional levels.

The 2021 INSS collected data from 514 regencies/cities across Indonesia, involving 14,889 census blocks and 153,228 children under five. This comprehensive dataset has been integrated with the National Socioeconomic Survey. Data was collected by trained enumerators with background in nutrition and following strict health protocols, including electronic records, disinfection of instruments, and personal protective equipment use. Additionally, 61 technical assistants across five regions ensured scientific, ethical, and health protocol compliance during data collection. This data was then processed into national, provincial, and district/city achievements.

This observational, analytical, cross-sectional study used individual secondary data from the Indonesian Nutritional Status Survey in 2021 in East Nusa Tenggara Province. Data were collected with the official permission of the Health Development Policy Agency, Ministry of Health. This study included 7,835 children aged 0–59 months living in the dryland area of the archipelago of East Nusa Tenggara Province. The primary outcome was stunting, defined by the WHO-Anthro program as a height-for-age z-score index value of less than -2 standard deviations. Other independent variables included in the study consisted of child and household factors. Child-related factors were categorized into three main categories: (1) child characteristics, including age, sex, birth weight, length at birth, and ownership of a birth certificate; (2) disease history, covering conditions like acute respiratory infection (ARI), diarrhea, and worm infections; and (3) health behavior, focusing on health integrated post-visit, breastfeeding practices, and the provision of supplementary feeding. The household factors include toilet type, national or local government health insurance ownership, *Kartu Keluarga Sejahtera (KKS)* card ownership, and residential areas. These factors and the specific characteristics of children under five are treated as explanatory variables, with their operational definitions provided below:

- **Low birth weight:** Children born weighing less than 2,500 grams.
- **Birth length:** Length at birth less than 48 centimeters.
- **Birth certificate ownership:** Children under five years who have a birth certificate.
- **Exclusive breastfeeding under 6 months:** Infants 0–5 months old exclusively breastfed.
- **Health integrated post visit:** Pregnant women and/or mothers of children under five who visit health integrated post to monitor the health of mothers and children.
- **Supplementary feeding:** The provision of extra food to children or families exceeding the standard portions of their usual diet.
- **Acute respiratory infection:** An infectious disease caused by viruses or bacteria, characterized by fever and symptoms such as sore throat, difficulty swallowing, hacking cough, or cough with phlegm.
- **Diarrhea:** Three or more liquid stools in 24 hours, which take the shape of the container they are in.
- **Worm infection:** Infestation with parasitic worms.
- **Vitamin A supplementation:** Receiving vitamin A capsule every 6 months (February and August) for infants ≥6 months old (red capsule (100.000 IU) for 6-11 months).
- **Basic immunization:** The number of children who received at least the basic immunization.
- **Toilet ownership:** Indicates whether the respondent has facilities for the disposal of human urine and feces.
- **Type of latrine/toilet:** Type of facilities used for disposal of human urine and feces (Gooseneck toilet, *Plengsengan* without a lid, *Plengsengan* with a lid, *Cemplung* without a lid, *Cemplung* with a lid, others).
- **National or local government health insurance ownership:** Respondents with national or local government health insurance.
- **Prosperous family card ownership:** Indicates possession of a Prosperous Family Card by the respondent.
- **Residence areas:** Refer to the type of district where people live, distinguishing between urban and rural areas.

### Population and subjects

The INSS involved 153,228 households with children under five years old across 14,889 census blocks. The study specifically targeted households with children under five and their mothers or caregivers who participated in the survey in 2021. The subjects were the caregivers of the infants and also the infants whose mothers participated in the study. The inclusion criteria were: 1) residing in East Nusa Tenggara Province, Indonesia, 2) caregiver of the infant and the infant whose mother was interviewed in the survey, and 3) willing to participate in the study. Exclusion criteria included families that relocated outside East Nusa Tenggara Province or could not be contacted.

### Sampling procedure

The sampling procedure of this study was based on the sampling procedure of the previous national study called the National Socio-Economic Study in March 2021. Initially, 22 districts were chosen, and from each village within these districts, a minimum proportionate number of households were chosen as potential participants using stratified two-stage sampling.

### Data analysis

Data analysis included data coding, editing, entry, and cleaning. The univariate, bivariate, and multivariate analyses were performed with the STATA 14 program using Complex Survey Analysis. First, the univariate analysis was performed to obtain an overview of the frequency distribution. Then, bivariate analysis was performed using the chi-square test with a significance level (*P* = 0.05) and 95% confidence interval (CI) to assess the relationship between child-specific factors (characteristics of children under five, disease history, and health behavior history) and household factors. Furthermore, multivariate analysis was performed using logistic regression to investigate the impact of several independent variables on stunting incidence.

## RESULTS

The analysis of the nutritional status of children aged 0 to 60 months, based on Height-for-Age Z-scores (HAZ), revealed critical insights into their growth patterns and potential factors contributing to stunted growth. The gender distribution among children aged 12 to 47 months (± 20%) was nearly equal, with females comprising 50.48% and males 49.52%. The data also showed that the majority (57.47%) of children under five did not have a birth certificate at birth, although they mostly had normal birth weight (83.35%) and length (75.55%). The data further indicated that during their early years, most toddlers encountered several health issues, such as infectious diseases (95.63%), diarrhea (85.96%), and helminthiasis (98.32%) (Table 1). Most mothers (93.77%) used the Posyandu (Integrated Services Post) for regular health monitoring of their children, contributing to a high rate of full immunization (61.49%) and appropriate vitamin A supplementation (76.38%). However, more than 72.12% of children under five did not receive adequate or complete exclusive breastfeeding. Moreover, interviews with mothers of children under five revealed that 79.70% of children who were malnourished were not involved in a supplementary feeding program.

**Table 1:** Characteristics of children in the dryland area, NTT Province 2021

Characteristics	*n*	%
**Age**		
0-5	683	9.09
6-11	959	12.12
12-23	1,565	19.95
24-35	1,606	20.73
36-47	1,615	20.32
48-60	1,407	17.80
**Gender**		
Male	3,807	49.52
Female	4,028	50.48
**Birth Certificate**		
Yes	3,522	42.53
No	4,313	57.47
**Low Birth Weight**		
No	6,815	86.35
Yes	1,020	13.65
**Body Length**		
Normal	5,919	75.55
Not Normal	1,916	24.45
**Upper Respiratory Infection**		
Yes	7,479	95.63
No	356	04.37
**Diarrhea**		
Yes	6,751	85.96
No	1,084	14.04
**Worms**		
Yes	7,702	98.32
No	133	01.68
Posyandu (*n* = 7768)*		
Yes	7,366	93.77
No	402	06.63
Immunization (*n =* 2424)*		
Yes	1,408	61.49
No	1,016	38.51
Vitamin A (*n* = 2424)*		
Yes	1,914	76.38
No	510	23.17
Exclusive Breastfeeding (*n* = 7347)**		
Yes	2,034	27.88
No	5,313	72.12
Supplementary Feeding (*n* = 7347)**		
Yes	1,655	20.03
No	5,692	79.70

* Data source from NTT Provincial Central Statistic Agency for 2017–2021 ** Data source from Indonesian Ministry of Health Data Center for 2017–2021

Most families of children under five lived in rural areas and did not have basic health insurance (78.44%) or family welfare cards (74.98%) (Table 2). However, most families of children under five (88.99%) already had a healthy toilet with a type of Gooseneck toilet (82.77%).

**Table 2:** Household characteristics in dry land, NTT Province 2021

Household characteristics	*n*	%
**Ownership of Latrines**		
Yes	6,980	88.99
No	855	11.01
**Type of toilet**		
Gooseneck	6,349	82.77
*Plengsengan* uncovered	437	4.70
*Plengsengan* covered	251	3.06
*Cemplung* uncovered	332	3.95
*Cemplung* covered	98	1.09
Others	368	4.42
**Ownership of JKN/ Jamkesda**		
Yes	1,820	21.56
No	6,003	78.44
**Ownership of KKS**		
Yes	2,218	25.02
No	5,617	74.98
**Area of Residence**		
Urban	1,190	14.98
Rural	6,645	85.02

Table 3 shows that age was significantly associated with stunting (*P* < 0.05) with a risk of 1.38–3.23 times. Additionally, factors such as exclusive breastfeeding, low birth weight, birth length, and ownership of birth certificates were also associated with the incidence of stunting (*P* < 0.05), with the risk being 1.19 to 1.76 times greater. On the other hand, factors including gender, infectious diseases, diarrhea incidence, frequency of Posyandu visits, vitamin A supplementation, and participation in supplementary food programs were correlated with stunting incidence. These factors can potentially act as protective elements, reducing the likelihood of stunting by a factor of one.

**Table 3:** The relationship between characteristics of children and stunting

Variables	Nutrition Status				*n*	%	*OR (CI 95%)	*P*
	No stunting		Stunting					
	*n*	%	*n*	%				
**Age**								
0-5	568	83.1	115	16.9	683	100	Ref	1.00
6-11	734	76.5	225	23.5	959	100	1.38 (1.02-1.86)	0.019
12-23	972	62.1	593	37.9	1,565	100	2.75 (2.11-3.59)	0.000
24-35	922	57.4	684	42.6	1,606	100	3.23 (2.51-4.16)	0.000
36-47	929	57.5	686	42.5	1,615	100	3.08 (2.39-3.98)	0.000
48-60	847	60.2	560	39.8	1,407	100	2.86 (2.19-3.73)	0.000
**Gender**								
Male	2,303	60.5	1,504	39.5	3,807	100	Ref	1.00
Female	2,682	66.6	1,346	33.4	4,028	100	0.77 (0.66-0.90)	0.001
**Possession of Birth Certificate**								
Yes	2,342	66.5	1,180	33.5	3,522	100	Ref	1.00
No	2,644	61.3	1,669	38.7	4,313	100	1.25 (1.12-1.40)	0.000
**Low Birth Weight (LBW)**								
No	4,455	65.4	2,360	34.6	6,815	100	Ref	1.00
Yes	528	51.7	492	48.3	1,020	100	1.76 (1.49-0.68)	0.000
**Body Length**								
Normal	3,819	64.5	2100	33.5	5,919	100	Ref	1.00
Not Normal	1,157	60.4	759	38.7	1,916	100	1.19 (0.009-1.04)	0.000
**Upper Respiratory Infection**								
Yes	4,396	58.78	3,083	41.22	7,479	100	Ref	1.00
No	229	64.28	127	35.72	356	100	0.79 (0.66-0.95)	0.001
**Diarrhea**								
Yes	3,614	53.53	3137	46.67	6,751	100	Ref	1.00
No	693	63.97	391	36.03	1,084	100	0.65 (0.51-0.83)	0.000
**Worms**								
Yes	4,060	52.72	3,642	47.28	7,702	100	Ref	1.00
No	85	63.7	48	36.3	17	100	0.64 (0.37-1.08)	0.0936
Posyandu (*n* = 7,768)*								
Yes	4,618	62.7	2748	37.3	7,366	100	Ref	1.00
No	293	72.89	109	27.11	402	100	0.63 (0.47-0.84)	0.008
Immunization (*n* = 2,424)*								
Yes	927	65.82	481	34.18	1,408	100	Ref	1.00
No	595	58.6	421	41.4	1,016	100	1.36 (1.12-1.65)	0.002
Vitamin A (*n* = 2,424)*								
Yes	1,126	58.81	788	41.19	1,914	100	Ref	1.00
No	393	77.04	117	22.96	510	100	0.43 (0.32-0.57)	0.000
**Exclusive Breastfeeding (*n* = 7,347)**								
Yes	1,388	68.26	646	31.74	2,034	100	Ref	1.00
No	3,179	59.83	2,134	40.17	5,313	100	1.44 (1.24-1.68)	0.000
**Supplementary Feeding (*n* = 7,347)**								
Yes	855	51.67	800	48.33	1,655	100	Ref	1.00
No	3,692	64.86	2000	35.14	5,692	100	0.58 (0.49-0.68)	0.000

* OR, Odds ratio

Most family characteristic factors were associated with stunting (Table 4). These factors were ownership of latrines and the types of latrines used, ownership of a basic health insurance card, and region of residence (*P* < 0.05), with a risk opportunity of up to 1.36–1.81 times greater. Conversely, owning a family card was also linked to stunting incidence but as a protective factor, reducing the likelihood of stunting by 0.61 to 0.80 times.

**Table 4:** The relationship between characteristics of household and stunting

Variables	No Stunting		Stunting		*OR (CI 95%)	*P* value
	*n*	%	*n*	%		
**Ownership of Latrines**						
Yes	4,489	64.31	2,491	35.69	Ref	1.00
No	488	57.03	367	42.97	1.36 (1.16-1.59)	0.000
**Type of toilet**						
Gooseneck	4,186	65.93	2,163	34.07	Ref	1.00
*Plengsengan* uncovered	239	54.68	198	45.32	1.60 (1.26-2.04)	0.000
*Plengsengan* covered	137	54.44	114	45.56	1.62 (1.18-2.23)	0.003
*Cemplung* uncovered	172	51.68	160	48.32	1.81 (1.27-2.58)	0.001
*Cemplung* covered	59	60.23	39	39.77	1.27 (0.85-1.93)	0.244
Others	199	54.18	169	45.82	1.63 (1.31-2.05)	0.000
**Ownership of JKN/ Jamkesda**						
Yes	1,333	73.24	487	26.76	Ref	1.00
No	3,652	60.84	2351	39.16	1.76 (1.47-2.11)	0.000
**Ownership of KKS**						
Yes	1,267	57.14	951	42.86	Ref	1.00
No	3,687	65.64	1930	34.36	0.69 (0.61-0.80)	0.000
**Area of Residence**						
Urban	676	56.78	514	43.22	Ref	1.00
Rural	3,620	54.47	3025	45.53	1.69 (1.47-1.94)	0.000

COR, Crude odds ratio

The multivariate analysis showed that age, gender, possession of a birth certificate, low birth weight, body length at birth, supplementary feeding, ownership of uncovered and uncovered pit latrines, ownership of basic health insurance, ownership of Prosperous Family Card, and area of residence were jointly associated with the incidence of stunting (*P* < 0.05) in the dryland islands of East Nusa Tenggara province from 2017-2021 with a risk of 1.26–2.85 times greater (Table 5).

**Table 5:** Determinants of stunting in dry land, NTT Province 2021

Child and Household Characteristics	*P* value	*AOR (CI 95%)
Age (X1)	0.000	2.856 (1.73-4.72)
Gender (X2)	0.000	0.73 (0.63 - 0.84)
Possession of Birth Certificate (X3)	0.000	1.26 (1.11 – 1.43)
Low Birth Weight Babies (X4)	0.000	1.90 (1.54-2.34)
Body Length (X5)	0.000	1.17 (1.01-1.36)
Upper Respiratory Infection (X6)	0.171	0.88 (0.71 – 1.06)
Diarrhea (X7)	0.092	0.800 (0.62 – 1.03)
Worms (X8)	0.197	0.75 (0.48 – 1.16)
Posyandu (X9)	0.104	0.78 (0.58 – 1.05)
Exclusive Breastfeeding (X10)	0.834	1.02 (0.82 – 1.27)
Supplementary Feeding (X11)	0.000	0.73 (0.62 – 0.87)
Ownership of Latrines (X12)	0.593	1.07 (0.84 – 1.35)
Type of toilet (X13)		
*Plengsengan* uncovered (X14)	0.016	1.38 (1.06 – 1.80)
*Plengsengan *covered (X15)	0.109	1.30 (0.94 – 1.80)
*Cemplung *uncovered (X16)	0.042	1.50 (1.01 – 2.23)
*Cemplung *covered (X17)	0.913	1.02 (0.65 – 1.62)
Others (X18)	0.047	1.42 (1.00 – 2.02)
Ownership of Basic Health Insurance (X19)	0.000	1.64 (1.37 – 1.97)
Ownership of Prosperous Family Card (X20)	0.000	0.75 (0.64-0.86)
Area of Residence (X21)	0.001	1.31(1.11 – 1.53)

* AOR, Adjusted odds ratio

The logistic linear regression model was obtained (Table 6):

**Table 6:** Partial analysis result

	Var. Independent	β	S.E	Wald	df	Sig.	Exp. (B)	95% C.I. For EXP (B)	
								Low	High
**Step.1**	X1	-0,964	0,442	0.120	1	0,000	2,86	1,73	4,72
	X2	-0,755	0,451	1,321	1	0,000	0,73	0,63	0,84
	X3	-0,125	0,625	2,590	1	0,000	1,26	1,11	1,43
	X4	1,757	0,625	5,342	1	0,000	1,90	1,54	2,34
	X5	-0,342	0,021	9,833	1	0,000	1,17	1,01	1,36
	X11	2,067	0,625	6,656	1	0,000	0,73	0,62	0,87
	X14	-2,310	0,456	3,241	1	0,016	1,38	1,06	1,80
	X16	-0,119	0,444	1,899	1	0,042	1,50	1,01	2,23
	X19	6,430	0,625	3.251	1	0,000	1,64	1,37	1,97
	X20	5,551	0,459	7,569	1	0,000	0,75	0,64	0,86
	X21	0,188	0,482	5,855	1	0,000	1,31	1,11	1,53
	Constant	2,639	0,995	15.995	1	0,001	2,33	-	-

$$\begin{array}{l} L(x)=2,639-0,964x1-0,755x2-0,125x3+1,757x4-\\ 0,342x5+52,067x11-2,310x14-0,119x16+6,430x19+\\ 5,551x20+0,188x21 \end{array}$$

Multiple logistic regression equation model:


$$\begin{array}{l} p=\frac{Exp(2,639-0,964-0,755-0,125+1,757-}{1+Exp(2,639-0,964-0,964-0,755-0,125+1,757}\\ \frac{0,342+2,067-2,310-0,119+6,430+5,551+0,188}{-0,342+2,067-2,310-0,119+6,430+5,551+0,188} \end{array}$$

$$p=\frac{Exp\left(15.742\right)}{1+Exp\left(15.472\right)}$$

The result showed that the chance of a child experiencing stunting with the above characteristics was 0.98 or 98%.

## DISCUSSION

The results showed a decrease in the prevalence of stunting in the province of NTT, where in 2021, there was a significant decrease of 3.02% compared to 2018 (39.51%) and 7.33% compared to 2019 (43.82%). However, most regencies/cities in the NTT province still have stunting rates above 30%. The three regencies/cities with the highest stunting rates were South Central Timor Regency, North Central Timor Regency, and Alor Regency. In South Central Timor Regency, for instance, the 2020 data from the local government highlighted significant socioeconomic challenges, with 37,320 individuals living in extreme poverty out of a total population of 455,410. In addition, only 60.04% or 69,602 households had proper sanitation. These conditions are identified as significant contributors to the community’s health vulnerability.

The prevalence of stunting, which exceeded 40%, indicates a public health problem that requires immediate intervention. The multivariate analysis revealed significant differences across various characteristics of children under five — such as gender, birth certificate ownership, low birth weight, and body length — with age being the only non-significant factor in the 6–11-month group. Children aged 36–47 months had a higher likelihood of stunting compared to those aged 0–5 months. These findings align with a study from Rwanda, which also identified an increased risk of stunting with age [4]. Research indicates that children aged 6–23 months have a lower risk compared to those aged 24–59 months. Similarly, a study conducted in Dale Woreda, Southern Ethiopia, highlighted that a child's age significantly influences the risk of stunting. Particularly, children who experience stunting before the age of two face substantial challenges in overcoming chronic nutritional deficiencies due to the entrenched nature of malnutrition at this critical developmental phase [5].

Furthermore, the study indicated that male participants were more susceptible to stunting, corroborating findings from the Mozambique Region, which found that boys in the 0–59 month age group were twice as likely to experience stunting [6]. Boys were more likely to experience malnutrition from the beginning of the fetal period, which can be related to socioeconomic factors. In addition, boys spend more time outside the home, playing with other boys, resulting in greater energy expenditure and exposure to infectious diseases [7].

The analysis also revealed that toddlers born with LBW were 1.76 times more likely to experience stunting compared to those with regular birth weights. Consistent with multiple studies, LBW is identified as a primary risk factor for stunting [8-10]. Children with LBW are at high risk of experiencing slower linear growth than children with normal LBW [11]. In addition to birth weight, children under five with abnormal birth length (<48 cm) also had a 1.17 times chance of experiencing stunting compared to toddlers with normal body length. This condition underscores the importance of fetal growth, which can be influenced by genetic factors and maternal nutritional intake during pregnancy. LBW, signaling poor nutritional status from pregnancy, has a profound impact on fetal development and is a critical factor in the occurrence of stunting [8,12]. This malnutrition can restrict the supply of nutrients through the placenta, affecting fetal growth [13-15]. Consequently, children born with LBW are highly likely to experience stunted growth and associated health issues in their early years, illustrating the significant role of prenatal nutrition in preventing stunting [8,16-20].

The bivariate analysis highlighted significant differences in the incidence of childhood illnesses such as ARI and diarrhea, except for helminthiasis, in relation to stunting. However, multivariate analysis showed that the history variable had no relationship to stunting. This outcome contrasts with findings from studies in Kupang and Timur Tengah Utara Regencies, NTT, where a notable link between infectious disease history and stunting was observed. In those studies, children under five with a history of infectious diseases were found to be three times more likely to experience stunting, highlighting the significant impact of illnesses like ARI, diarrhea, and pneumonia on child health and growth [21,22].

Stunting is intricately connected not only to growth challenges but also to increased susceptibility to diseases. Factors such as exposure to smoke within the household and living in traditional houses (Loppo in NTT) with poor ventilation and dirt floors can significantly increase the risk of ARI and pneumonia. Additionally, the scarcity of clean water exacerbates the risk of diarrheal diseases, further hindered by the lack of clean living habits, such as regular handwashing and sanitation practices. These health challenges critically contribute to the higher incidence of stunting, with infectious diseases diminishing children's appetite and compromising their immune systems, thereby exacerbating malnutrition [5,23,24].

Furthermore, the analysis revealed that while health behaviors like receiving supplementary feeding (PMT) were associated with stunting, breastfeeding and attendance at Posyandu (integrated health services post) did not show a significant impact on stunting rates. The rate of exclusive breastfeeding for infants aged 0-5 months in NTT was low (75%) and remains below the national target (93%), suggesting a potential risk factor for stunting. This is similar to another study where the exclusive breastfeeding factor played a major role in determining stunting [3]. However, certain studies, such as research conducted in Kupang Regency, have reported no significant relationship between exclusive breastfeeding and the incidence of stunting. This study found that a considerable majority of children under five (66.1%) received exclusive breastfeeding adequately during the first six months of life or had a history of exclusive breastfeeding [21]. Despite varying findings across studies, the importance of exclusive breastfeeding — recommended by the Ministry of Health for the first year of life and complemented by solid foods from six months, with WHO guidelines advocating for continuation up to two years — is underscored as a key determinant in addressing stunting [25-28]. Observational and interview data from various studies suggest several reasons behind the low rates of exclusive breastfeeding in NTT, including prevailing beliefs that exclusive breastfeeding is not mandatory, mothers experiencing nipple pain or injuries, insufficient milk production, and cultural or familial restrictions against breastfeeding. These factors significantly contribute to the challenge of increasing exclusive breastfeeding coverage in the region. Furthermore, the bivariate results showed a relationship between a history of immunization and vitamin A supplementation with the incidence of stunting. Immunization plays a crucial role in enhancing the body's immunity, protecting children from infections that can compromise their appetite and nutrient intake, ultimately disrupting growth hormone activity and hindering growth. The Indonesian Basic Health Survey (Riskesdas 2018) revealed that complete immunization coverage among children under five in NTT was notably low at 51.6%, falling below the national average of 57.9%. This deficit in immunization rates suggests that children who are not fully immunized in NTT are at higher risk of contributing to the stunting statistics in the region [29]. Given the geographical challenges in the NTT region and the limited access to healthcare services and support from health workers, several factors contribute to the low immunization coverage. These include the primary concern of mothers of children under five with agricultural work, lack of financial resources and transportation, distant locations of immunization sites, and insufficient awareness about immunization schedules. The multivariate analysis further indicated that household characteristics such as the use of uncovered latrines (*plengsengan* and *plunger*), lack of health insurance (JKN/Jamkesda card), ownership of KKS, and living in rural areas were associated with a higher incidence of stunting. Proper sanitation facilities, such as toilets with lids, septic tanks, or wastewater treatment systems, significantly reduce exposure to infectious diseases like diarrhea, a risk factor for stunting [30]. This study aligns with findings from the Cicalengka Health Center in Bandung Regency, showing a higher prevalence of stunting among children using inadequate sanitation facilities. The better the condition of the latrines, the lower the risk of stunting [31]. The results of this study indicate that toddlers who do not have a JKN/Jamkesda card experience a higher incidence of stunting (39%).

The results of this study showed that toddlers who lived in rural areas had a higher chance of experiencing stunting, which can be attributed to socioeconomic and cultural factors. Data from the Indonesian Central Statistic Agency (BPS) underscore the economic challenges faced by the residents of NTT, with over 21% of the population living below the poverty line. This translates to approximately 1146.32 individuals per Regency/City in East Nusa Tenggara Province, with a daily per capita income of IDR 403,005. Most individuals in the NTT province work in the agricultural sector. However, economic analysis indicates that this sector contributes only 28.89% to the Gross Regional Domestic Product (GRDP) at Current Prices in NTT Province [32]. This disparity has direct implications for the purchasing power of families with children under five, impacting the nutritional adequacy of young children.

Furthermore, the economic status of pregnant women has been shown to influence maternal health, which in turn correlates with the occurrence of stunting in newborns [33-35]. Moreover, mothers engaged in farming often have limited time for childcare and meal preparation at home, potentially affecting children's dietary patterns and nutritional intake [36,37]. The results showed that food diversity, dietary patterns, and the amount of food were associated with the incidence of stunting [38-40].

## CONCLUSION

The results showed that the incidence of stunting in children under five in the archipelagic dryland region of East Nusa Tenggara Province was very high at 36.49%. Multivariate analysis identified a significant relationship between stunting and the children's characteristics (age, sex, low birth weight, body length, and birth certificate ownership), health behavior (specifically, receiving PMT), and household characteristics (such as toilet type, ownership of JKN/Jamkesda card, Prosperous Family Card, and residential area). However, no significant relationship was observed between a history of childhood illnesses (ARI, diarrhea, and helminthiasis) and stunting incidence. There is a need to increase the stunting control program in a convergent and holistic way at the individual and family level in the archipelagic dryland area of East Nusa Tenggara Province by involving the government, community, academia, entrepreneurs, and the media.

## Personal thanks

We would like to thank the Health Policy Agency, Ministry of Health Republic of Indonesia, BPS - Statistics Indonesia of East Nusa Tenggara Province, Development Planning Agency at Sub-National Level of East Nusa Tenggara Province, Team Work Stunting of East Nusa Tenggara Province, and local government for their support.
